# SlowFaster, a user-friendly program for slow-fast analysis and its application on phylogeny of *Blastocystis*

**DOI:** 10.1186/1471-2105-9-341

**Published:** 2008-08-15

**Authors:** Martin Kostka, Magdalena Uzlikova, Ivan Cepicka, Jaroslav Flegr

**Affiliations:** 1 Department of Parasitology, Faculty of Science, Charles University, Vinicna 7, 128 44 Prague, Czech Republic; 2 Department of Anatomy and Physiology of Farm Animals, Faculty of Agriculture, University of South Bohemia in Ceske Budejovice, Studentska 13, 370 05 Ceske Budejovice, Czech Republic; 3 Department of Zoology, Faculty of Science, Charles University, Vinicna 7, 128 44 Prague, Czech Republic; 4 Department of Philosophy and History of Science, Faculty of Science, Charles University, Vinicna 7, 128 44 Prague, Czech Republic

## Abstract

**Background:**

Slow-fast analysis is a simple and effective method to reduce the influence of substitution saturation, one of the causes of phylogenetic noise and long branch attraction (LBA) artifacts. In several steps of increasing stringency, the slow-fast analysis omits the fastest substituting alignment positions from the analysed dataset and thus increases its signal/noise ratio.

**Results:**

Our program SlowFaster automates the process of assessing the substitution rate of the alignment positions and the process of producing new alignments by deleting the saturated positions. Its use is very simple. It goes through the whole process in several steps: data input – necessary choices – production of new alignments.

**Conclusion:**

SlowFaster is a user-friendly tool providing new alignments prepared with slow-fast analysis. These data can be used for further phylogenetic analyses with lower risk of long branch attraction artifacts.

## Background

The long branch attraction (LBA) artifact [1] still remains one of important causes of biases and mistakes in phylogenetic analyses of sequence data [2]. LBA causes taxa with long branches to be artifactually grouped with or attracted to other long branched taxa (i.e., fast evolving taxa or taxa evolving for a long time separate from other groups, e.g. outgroups). An important source of LBA is substitution saturation of positions in alignment (the term "mutational saturation" is also used, although it is not correct in this context). It would be ideal to have positions that underwent a single or a few changes during evolution, but many positions in real alignments are subject to multiple substitutions. This subset of rapidly evolving positions is the source of stochastic noise rather than useful signal. However, these saturated positions are responsible for a major part of information used in phylogenetic analyses [3], which could confuse most of the tree-reconstructing methods. Because there are only four possible states for nucleic acid data (20 for amino acids), it is probable that a part of saturated positions will evolve randomly – convergently into the same state. It could then be erroneously judged as a synapomorphy. LBA can thus be a major problem especially in maximum parsimony, but occurs also in other analyses [4]. Maximum likelihood can, under an appropriate model of evolution, deal better with saturated positions, but datasets containing sites with different rates of substitution across the tree (covarion-like) may still be problematic [5]. Besides LBA, a high level of saturation in the dataset may cause signal simply to be overwhelmed by noise at least at some points of the tree topology. Such nodes could be resolved incorrectly or (at least) with a low statistical support.

It has been shown that in real alignments, LBA can be a major problem [2]. An effective way to estimate and reduce the effect of substitution saturation and LBA is removal of fast-evolving data. One such method is slow-fast analysis of the dataset [6]. The positions of the alignment are divided into several classes according to their substitution rate (estimated within *a priori *defined monophyletic groups). Several new alignments are then created, which contain only positions with a substitution rate lower than several thresholds, ranging from maximum to minimum rate. Thus the signal/noise ratio of the alignments successively increases, however, on the expense of amount of positions included in the alignment. Technically, the Slow-Fast method needs some input tree topology to work with. The topology must be provided by primary phylogenetic analysis of the dataset or by another independent method. This topology is needed for recognition of some monophyletic groups (whose relative positions on the tree is not necessary to know before slow-fast analysis). Maximum parsimony is then used to determine the number of changes for each position within the monophyletic subgroups. Substitution rates assigned to positions are thus independent from interrelationships among the monophyletic groups, and therefore, these interrelationships may in turn be investigated without the fear of circularity. When each position is assigned its number of changes, those with the highest substitution rate are gradually omitted from new alignments. The following phylogenetic analyses of these new datasets (starting from the dataset containing the positions with the highest substitution rate) then provide results based on decreasing number of sequence data, however, with decreasing risk of artefactual groupings of long branches. There are several good examples of successful use of slow-fast analysis, see e.g. [6-11].

Although the slow-fast analysis is relatively powerful and very simple in principle, it is quite demanding when one wants to determine the number of changes for individual alignment positions (e.g., with the help of PAUP [12], using the "describetree" command) and the manual procedure of deleting of positions by editing the dataset is especially very time consuming. We believe that this is one of the most important reasons why this method is used relatively scarcely. Clearly, a computer program that provides this evaluation of positions and which produces new alignments would be handy. To our knowledge, the only software providing slow-fast analysis have been MUST [13]. MUST is a complex package, yet it still does not provide a quick and easily operated tool for this type of analysis. This is what our program SlowFaster does. It is a user-friendly tool to conduct slow-fast analysis and produce a set of new alignments without fast evolving positions. It have several additional functions. Note that another program for slow-fast analysis was presented recently [11].

## Implementation

SlowFaster was programmed in Borland Delphi and runs under MS Windows. Both the executable file [see Additional file 1] and the source code [see Additional file 2] are available as supplements. The program leads the user in several steps through the process of generating new datasets. Original alignment is loaded in FASTA, Phylip or NEXUS format. The program works with both nucleic acid and amino acid alignments and supports usual ambiguity coding. The topology needed for the recognition of monophyla is loaded as a tree in the Newick ("bracketed") format (PAUP users can use "savetree format = phylip" command to obtain tree in Newick format). After choosing the monophyletic groups by simply clicking on the branches of the depicted tree, parsimony is used to count the number of changes of every alignment position within the selected groups. Finally, new alignments are produced (in FASTA, Phylip or NEXUS format). Each of the new datasets has a number which is a threshold: positions with greater number of changes were omitted from this dataset. As the threshold gets lower and lower, the datasets contain fewer and fewer data because the more saturated positions were deleted from them. These datasets can be then further analysed to obtain phylogenies with a lower risk of LBA. During the whole process, there are hints shown in a window, telling the user what to do in the given step.

The software was tested thoroughly on several model datasets [see Additional file 3] and also on dataset of Hampl *et al*. [10]. In this latter case, we obtained the same new datasets with our program (Hampl *et al*. obtained them with the help of PAUP and through careful manual deletion of positions).

An interesting alternative to slow-fast method is using substitution rates estimated with maximum likelihood (ML). Although ML estimates are not implemented in SlowFaster, this program enables production of alignments without positions with high rates through the "Load changes" button. The rates can be counted in another software. E.g. Tree-Puzzle [14], if rate heterogeneity is selected, gives information on the rate category of each position in its outfile under "Combination of categories that contributes the most to the likelihood". These data can be simply copied in a file which is then loaded in SlowFaster. New alignments are then produced directly from these data. More generally, any sequence of any (even real) numbers can be loaded and the software will divide positions in rate categories (their number is specified by the user) based on these values.

The program also creates a log file which contains useful information, most notably groups used for changes counting, list of positions with certain number of changes and number of changes for all positions from the first one to the last.

## Results and discussion

### Sample Data

As an example, we analysed an alignment of 34 SSU rDNA sequences of 31 isolates of *Blastocystis *+ 3 outgroups. *Blastocystis *is an unusual protist, a sister group of slopalinids (used as the outgroup) within the group of stramenopiles. See e.g. [15,16] for a review of *Blastocystis*. Although these nonflagellated, multinucleated gut commensals comprise a single genus, their SSU rDNA phylogeny shows clearly that they are rather long branched taxa in comparison to other stramenopiles. Their branches are even longer than, for example, branches separating classes of autotrophic stramenopiles. This group is therefore suspected of a high level of substitution saturation. We sequenced SSU rRNA genes of five *Blastocystis *isolated from tortoises to improve taxon sampling by increasing the number of non-mammalian and non-bird isolates in the analysis (the vast majority of *Blastocystis *sequences available in GenBank are from bird or mammalian isolates). The accession numbers of the five new sequences (GERA3b, GERA3a, GECA2, KINIX2 and GEPA2) are [GenBank:EF209016], [GenBank:EF209017], [GenBank:EF209018], [GenBank:EF209019] and [GenBank:EF209020], respectively.

**Figure 1:**
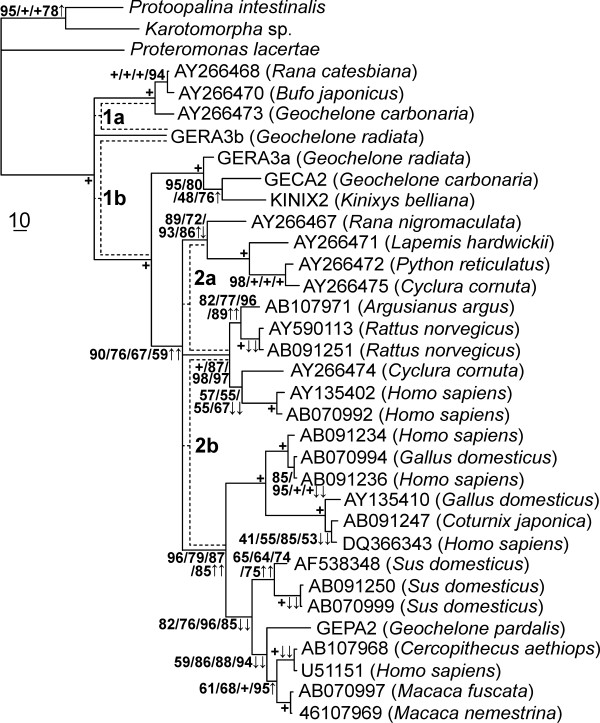
**MP tree of 31 *Blastocystis *isolates and 3 outgroups based on SSU rDNA sequences**. MP tree of 31 *Blastocystis *isolates (host in brackets) and 3 outgroups, based on SSU rDNA sequences. Bootstrap support values for four tree-reconstructing methods – ML, MP, LD and MD, respectively – are shown at the nodes. The symbol "+" is used for bootstrap support 99 and higher (in case only one "+" symbol is present, all methods scored such a high support). The effect of slow-fast analysis on nodes is represented by arrow symbols in the figure. Increase of an average bootstrap support by more than 10% of one and more than one tree-reconstructing method in two datasets (BlastS3 and S2) is marked with "↑" and "↑↑", respectively. Similarly, the decrease of bootstrap support is marked with "↓" and "↓↓" at the particular nodes. Bootstraps of other nodes did not change dramatically. Except for our five new isolates (GERA3A, GERA3B, GEPA2, GECA2, KINIX2), *Blastocystis *isolates are labeled with accession numbers of their SSU rRNA gene sequences.

The alignment was prepared with ClustalX [17] and ambiguous parts with many indels were deleted from the alignment in the program BioEdit [18]. The resulting alignment contained 1471 positions. PAUP 4.0β10 [12] was used to analyse the dataset employing maximum likelihood (ML), maximum parsimony (MP), the Fitch-Margoliash method with LogDet distances (LD) and maximum likelihood distances (MD). Appropriate models for maximum likelihood were chosen with the help of Modeltest [19]. The robustness of each obtained topology was tested by bootstrapping (1000 replicates for all methods except for ML, for which 100 replicates were used). Phylogenetic analyses resulted in the tree shown in fig. 1. Two deep nodes of the phylogeny were resolved with low bootstrap support and/or resolved differently by different methods and were therefore depicted and treated as unresolved trichotomies.

### Use of SlowFaster

At this time, our SlowFaster program was employed to perform the slow-fast analysis. First, the alignment used in our analyses was loaded via the "Load alignment" button. Then the tree topology shown in fig. 1 was loaded via the "Load tree" button. In typical slow-fast analyses, several monophyletic subgroups are chosen in this step. We decided to select the single subtree of all *Blastocystis *isolates. This arrangement was enabled by the fact that we were mostly interested in resolving the two nodes represented in the input tree by trichotomies. Assigning substitution rates to alignment positions was thus independent from the true topology of these nodes. When the *Blastocystis*-containing subgroup was chosen in the tree window of SlowFaster program, new datasets in NEXUS format were created by clicking the "New alignments" button. Also, alignments of the same length as these new datasets, but shortened by random deletion of positions, were prepared by checking the "jackknives" checkbox on the program screen. These were used to test whether the loss of informative positions influences decrease of bootstrap support of the resulting tree topology more than shortening the datasets itself. We did not use the "Weights" feature of the program. When this checkbox is checked, the algorithm will assign different weights to changes within different chosen monophyletic groups. Changes within smaller groups would have assigned greater weight (if group A is twice as taxon-rich as group B, changes within it will have half the weight of the weight of changes in group B). The impact of large monophyla is then not dominant just because they contain more taxa.

The maximum number of observed changes in a position of our alignment was 9. Thus, nine new alignments were created. They were labeled BlastoS8 down to BlastoS0, where the number is the threshold. BlastoS0 alignment was of course of no use in this particular case (the analysis with just one monophyletic group) as it contained only those positions that did not change during the evolution of *Blastocystis*. All other alignments were analysed phylogenetically by all four methods (ML, MP, LD, MD) and topologies of the 32 resulting trees were bootstrapped.

It is highly probable that in some point of the slow-fast analysis, the profit from diminishing noise is lower than the loss from diminishing information. To roughly estimate the effect of the lack of information, we used average values of bootstraps as a measure of reliability of the alignments [10]. We found that this average value drops suddenly for the alignment BlastoS1 which is therefore likely to suffer from lack of information and the resulting trees obtained from this dataset were not taken into account. To further prove this decision, "jackknifed" datasets of the same length but shortened by random deletion of position were also analysed. For each of eight datasets (Blasto_S1 to S8), ten of these randomly shortened datasets were analysed (80 alignments on the whole: Blasto_J1_1 to J1_10, J2_1 to J2_10, ... J8_1 to Blasto_J8_10). Within each dataset, the average value of bootstraps was determined and average of these averages for ten dataset of the same length were compared to average bootstrap value of the respective dataset resulting from slow-fast analysis. This comparison showed that the bootstrap values does not change much when analysing J8_x down-to J1_x datasets (e.g. all these average values ranged from 84.76 to 86.36 in ML analyses or from 90.15 to 91.5 in LD). On the contrary, the downfall of bootstraps was much more prominent in Blasto_S1 dataset when compared to Blasto_S2 – BlastoS8 datasets (e.g. 87.19 for original dataset, 86.90 for Blasto_S2, but 81.13 for Blasto_S1 in ML analyses, or 91.29 and 88.03 vs. 79.13, respectively, for LD).

Results concerning the two unresolved trichotomies are shown in Table 1. The isolate GERA3b grouped either with the basal branch of three reptile/amphibian isolates (1a, in fig. 1) or with the rest of *Blastocystis *(1b). In the original alignment, the former topology was very well supported by MP and LD, the latter was weakly supported by ML and MD. As the most saturated positions were deleted from alignment, the bootstrap support for topology "1a" decreased slightly in MP, but increased strikingly in MD and slightly in ML analysis (BlastoS1 not taken into account). The slow-fast analysis thus supports the "1a" topology. The second unresolved node concerned a branch of four reptile/amphibian isolates. Either it was basal to two major branches of mostly mammal/bird isolates (2b; weakly supported by ML and MP in the original alignment), or it grouped with one of them (2a; weakly supported by LD and MD). After the slow-fast method was applied, both LD and MD favored the first possibility with reasonable bootstrap support for S3 and S2 datasets. However, MP and ML were unable to decide on the two possibilities. We conclude that the "2b" topology is probably correct, although the certainty is not high. For other nodes, decrease/increase of their bootstrap support from datasets S3 and S2 is marked in fig. 1.

**Table 1:** Overview of results from slow-fast analysis of *Blastocystis *alignment

Dataset	Posit.	Length	1a				1b				2a				2b			
			
			ML	MP	LD	MD	ML	MP	LD	MD	ML	MP	LD	MD	ML	MP	LD	MD
**Untr**.	1471	1289		92	99		54			58	50	33					46	35
**S8**	1467	1250		93	99		51			54	45			30		34	43	
**S7**	1460	1187	57	96	99	54					42	34		36			37	
**S6**	1452	1121	62	96	99	48					48	35		38			38	
**S5**	1438	1026	61	91	97	55					54	42		34			35	
**S4**	1407	844	63	87	99	73					57			36		45	36	
**S3**	1371	674	59	82	97	85					-	38	67	73	-			
**S2**	1330	522	68	75	98	97					-	30	64	64	-			
**S1**	1258	343		49	92	90	71				-	-	90	76	-	-		
**S0**	1097	124																

For each dataset (the first column) ranging from untreated initial alignment (Untr.) to alignment BlastoS0, the number of alignment positions (Posit.) and the length of the most parsimonious tree (Length) are noted in the second and third columns, respectively. In the remaining columns is given the bootstrap support from the four tree reconstructing methods for four topologies of interest. In some cases (marked with a dash) the method was unable to decide between the given node and its alternative.

## Conclusion

Overall, the slow-fast analysis, provided by the program SlowFaster, proved to be a useful tool to solve uncertain phylogenies by increasing the signal/noise ratio. In the *Blastocystis *SSU rDNA tree it was able to make a choice among competing hypotheses and add more confidence in some other cases. Our software automates quite time-consuming slow-fast analysis.

## Availability and requirements

**Project name**: SlowFaster

**Project home page**: 

**Operating system**: MS Windows

**Programming language**: Borland Delphi

**Any restrictions to use by non-academics**: none

The software can be accessed through the project home page and its current version is included with the manuscript as an additional file.

## Authors' contributions

MK and JF designed the program and contributed bug fixes. MK developed the source code. IC and MU collected the data used as example and analysed them together with MK. These three authors contributed to writing the manuscript. All authors read and approved the final manuscript.

## Supplementary Material

Additional file 1 **SlowFaster**. This is the executable file of the application. Click here for file

Additional file 2 **Source code**. Zip archive containing Delphi source code of the program and additional Delphi files. Click here for file

Additional file 3 **Sample data**. Zip archive containing sample data – alignments in Phylip, FASTA and NEXUS format and tree files in Newick format. Click here for file
